# Relationship between psychological detachment from work and depressive symptoms: indirect role of emotional exhaustion and moderating role of self-compassion

**DOI:** 10.1186/s40359-023-01384-z

**Published:** 2023-10-18

**Authors:** Qinglu Wu, Tengfei Qi, Jun Wei, Amy Shaw

**Affiliations:** 1 Institute of Advanced Studies in Humanities and Social Sciences, Beijing Normal University, Zhuhai, China; 2 Department of Sociology, School of Government, Shenzhen University, Shenzhen, China; 3 Institute of Education, Tsinghua University, Beijing, China; 4 Department of Psychology, Faculty of Social Sciences, University of Macau, Macau, China

**Keywords:** Psychological detachment, Emotional exhaustion, Self-compassion, Depressive symptoms

## Abstract

The importance of psychological detachment during nonwork time has been emphasized, and its effect on depressive symptoms has been identified. However, the mechanism underlying this association remains to be elucidated. This study was conducted to identify the indirect role of emotional exhaustion in the association between psychological detachment and depressive symptoms and the moderating role of self-compassion in the relationship between emotional exhaustion and depressive symptoms on the basis of the recovery–engagement–exhaustion model and emotion regulation theory. Through an online survey, relevant data were collected from 727 university teachers (mean age, 37.65 ± 7.77 years; 38.5% men). Indirect and moderation effect were analyzed through structural equation modeling (Mplus). Psychological detachment was found to be associated with depressive symptoms through emotional exhaustion. Self-compassion moderated the association between emotional exhaustion and depressive symptoms. The association between emotional exhaustion and depressive symptoms was weaker among university teachers with high levels of self-compassion than among those with low levels of self-compassion. This study improves our understanding of the association between psychological detachment and depressive symptoms by identifying the emotional pathway and protective function of self-compassion. Interventions for improving mental health in work context should be designed considering psychological detachment and self-compassion to deal with work-induced emotional strain.

## Introduction

High levels of work stress have adverse consequences, including the impairment of emotional well-being (e.g., positive and negative affect), relational well-being (e.g., social undermining toward partner), and mental health (e.g., increased depressive symptoms) [1–4]. As a core recovery experience, psychological detachment has received substantial attention because mental disengagement during nonwork hours and leisure time helps individuals to detach from work pressure and recover during rest [5, 6]. Psychological detachment is positively associated with recovery, mental health, work engagement, and well-being [7–10] and buffers the negative effects of work-related stressors on work and mental health outcomes [11, 12]. Most studies focus on the antecedents (e.g., learning goal orientation and problem-solving rumination) and direct consequences (e.g., reduced insomnia and work engagement) of psychological detachment; a negative association has been reported between psychological detachment and depressive symptoms [13–16]. However, very few studies have been conducted to investigate the mechanism underlying the association between psychological detachment and outcomes (e.g., well-being and emotional rejection) [17–19]. How psychological detachment contributes to reducing individuals’ depressive symptoms requires further investigation. In the present study, we investigated the indirect role of emotional exhaustion and the moderating role of self-compassion in the association between emotional exhaustion and depressive symptoms on the basis of the recovery–engagement–exhaustion model and emotion regulation theory [10, 20].

The importance of recovery experiences has been emphasized in studies on the alleviation of job stress. Drawing on the positive psychology perspective, Newman and colleagues [5] proposed the DRAMMA model, which highlights the roles of detachment, recovery, autonomy, mastery, meaning, and affiliation in the relationship between leisure and subjective well-being. On the basis of the mood regulation and recovery, Sonnentag and colleagues [6] identified four recovery experiences (psychological detachment from work, relaxation, mastery, and control) and demonstrated their associations with various job stressors, strain reactions, and psychological well-being [21]. As a core recovery experience, psychological detachment from work has received considerable attention.

Psychological detachment refers to mental disconnection or disengagement from work (not thinking about job-related matters) and refrainment from job-related activities during nonwork time [22, 23]. Psychological detachment, which helps reduce the psychological strain resulting from stressful work situations [22], is associated with affective states, well-being, sleep quality, and psychological adjustment [7, 8, 24]. One important contribution of psychological detachment is its effect on depressive symptoms, which has been identified in different populations (e.g., managers and primary school teachers) [15, 25, 26]. Teachers who react negatively to imperfection are less likely to experience mental detachment from work, which leads to higher levels of depressive symptoms [15]. Furthermore, among managers, core self-evaluation can weaken the association between psychological detachment and depressive symptoms [25]. Although the association between psychological detachment and depressive symptoms has already been reported, the precise mechanism underlying this association is yet to be comprehensively understood [10, 27].

As a core component of burnout, emotional exhaustion may play an indirect role in the association between psychological detachment and depressive symptoms. Emotional exhaustion is a chronic state of physical and emotional depletion (e.g., emotional overextension and drain) resulting from excessive work or personal demands and a heavy workload [28–31]. Emotional exhaustion at work is an antecedent of depressive symptoms [32, 33]. Notably, emotional exhaustion mediates the longitudinal association between sleep disturbance and depressive symptoms among nursing students [28]. Informal caregivers with high levels of emotional exhaustion are more likely to be depressed and perpetrate physical violence toward care recipients [34]. Emotional exhaustion can be relieved by detachment from work. Psychological detachment has been demonstrated to exert a substantial effect on emotional exhaustion [7, 10] and it predicts changes of emotional exhaustion over time [8, 9].

The recovery–engagement–exhaustion model, proposed by Headrick and colleagues [10], supports the indirect role of emotional exhaustion in the association between psychological detachment and depressive symptoms. This model was developed on the basis of meta-analytic associations between recovery experiences, engagement, exhaustion, and outcomes in work and health domains. The model illustrates that two separate mechanisms (engagement and exhaustion pathways) link recovery experiences with work and health outcomes. In this dual-process model, work engagement and exhaustion mediate the effects of recovery experiences on outcomes. Of four recovery experiences (i.e., psychological detachment, relaxation, mastery, and control), only psychological detachment has substantive effect on exhaustion [10]. Reducing emotional exhaustion may translate the positive function of psychological detachment in ameliorating depressive symptoms. In university teachers, psychological detachment may alleviate emotional exhaustion, thus preventing the exacerbation of depressive symptoms.

As an effective emotional self-regulation strategy and a personal resource [35, 36], self-compassion may moderate the association between emotional exhaustion and depressive symptoms. Self-compassion refers to exhibiting positive and supportive responses toward oneself when encountering personal inadequacies or external difficulties in life [36, 37]. It includes compassionate self-responding (i.e., self-kindness, common humanity, and mindfulness) and reduced uncompassionate responding (i.e., reduced self-judgement, isolation, and over-identification). Individuals with high levels of self-compassion are more likely to treat themselves in a caring (rather than harsh) manner, view suffering as a shared (rather than isolated) human experience, and address negative thoughts and emotions in a balanced manner (rather than over-identifying with them). Self-compassionate individuals usually treat themselves with kindness and accept personal flaws when facing substantial work stress [38, 39]. They may believe that stressful work consequences (e.g., work-related rumination) are common experiences shared by individuals in the same circumstances. In addition, these individuals regulate their emotions and maintain a balanced view of their situation when facing work stress. Thus, self-compassion helps them to cope with the negative consequences of high work stress in a resilient manner.

Emotion regulation theory emphasizes the importance of regulating the magnitude and duration of emotional responses with activation purpose (e.g., promoting positive affect and alleviating negative affect), which is beneficial for individuals dealing with challenges in daily life [40]. As an emotional self-regulation strategy, self-compassion buffers the adverse effects of emotional stressors or strains on depressive symptoms [41–43]. The association of emotional stressors or strains (e.g., external shame and body shame) with depressive symptoms is weaker (or even nonexistent) among individuals with high levels of self-compassion than among those with low levels of self-compassion [42, 43]. Individuals who self-respond compassionately are more inclined to functionally address emotional stressors and strains. Treating themselves in a caring manner, maintaining emotional stability, and understanding the situation from a comprehensive perspective help these individuals avoid the adverse effects of negative emotional processes. For teachers, transitioning from work to home and disengaging from their professional role is difficult; high daily levels of emotional demand usually lead to maladaptive affective states [2, 24, 44]. Because self-compassion is a personal resource that can buffer the adverse effects of emotional stressors or strains on different outcomes, it may buffer the negative effect of work-related emotional exhaustion on depressive symptoms in university teachers.

**Fig. 1:**
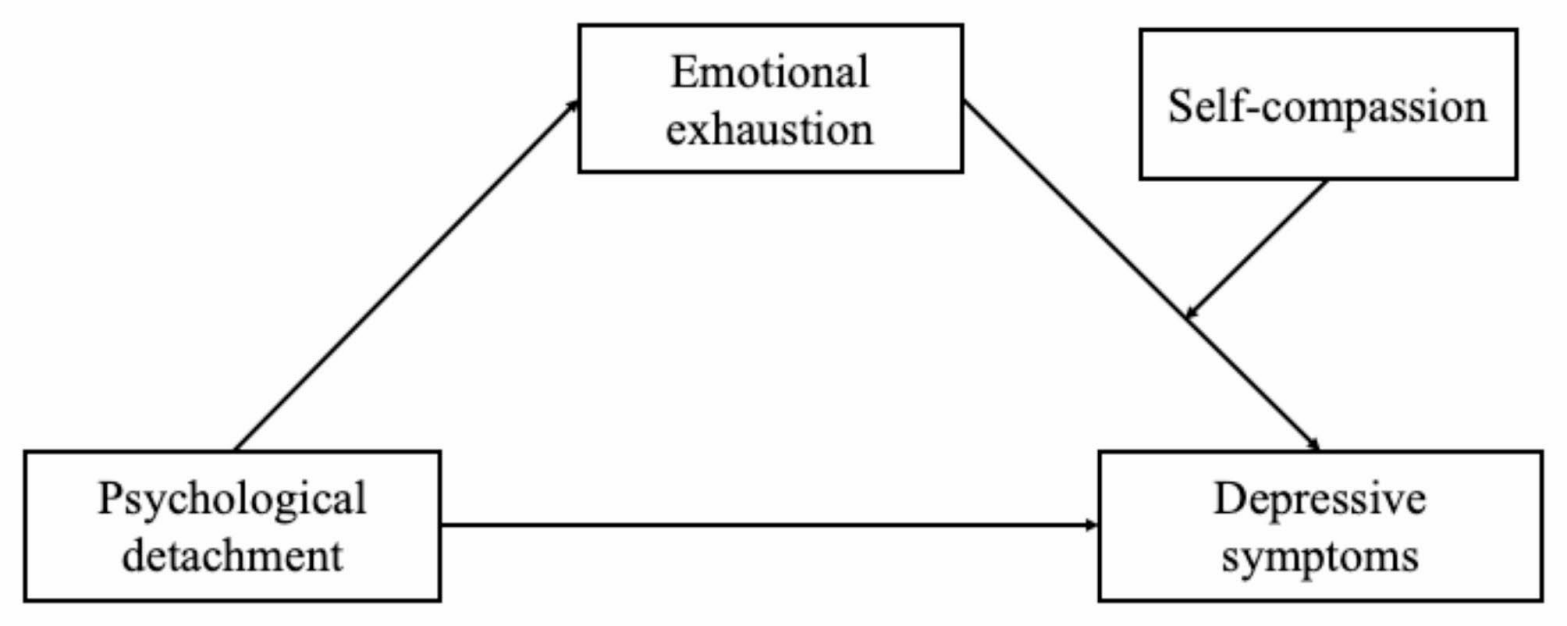
Hypothesized model

Although studies on the positive function of psychological detachment mainly focus on its antecedents (e.g., learning goal orientation), direct consequences (e.g., reduced insomnia and depressive symptoms), and its buffering effects in the association between work-related stressors on work and mental health outcomes [11–16], the mechanisms underlying the positive effects of psychological detachment on outcomes (e.g., well-being and job performance) remain unclear [27]. A limited number of studies have investigated how psychological detachment contributes to health outcomes and the mechanism underlying this association (for a meta-analysis, see [10]), which is key to understanding the function of psychological detachment and applying it in stress management. In the present study, we adopted the recovery-engagement–exhaustion model and emotion regulation theory [10, 20] and examined emotional exhaustion as an emotional pathway from psychological detachment to depressive symptoms and the moderating role of self-compassion in the association between emotional exhaustion and depressive symptoms among university teachers. We hypothesized that university teachers who had mental detachment from work during nonwork time would experience less emotional exhaustion and thereby low levels of depressive symptoms. We further hypothesized that the association between emotional exhaustion and depressive symptoms would be weaker among teachers with high levels of self-compassion than among those with low levels of self-compassion. The model hypothesized in the present study is shown in Fig. 1.

## Methods

### Participants and Procedure

Data used in the present study were obtained from a project on the relationship between work stress and psychosocial adjustment among Chinese university teachers. University teachers were recruited to participate in this project. Recruitment information was disseminated by authors and university teachers through social networks. Participants were informed about the study purpose, data confidentiality, and voluntary participation. They provided informed consent before completing the online survey. Qualifying participants received a small monetary reward (80 RMB; approximately US $ 11). The project was approved by the Research Ethics Committee of the School of Social Development and Public Policy at Beijing Normal University.

In total, 800 participants completed the online survey. After the exclusion of participants who failed attention check (*n* = 72) or whose affiliation was outside China (*n* = 1), the final sample included 727 participants (280 men). The sample comprised 269 (37%) teachers from national key institutions (211/985 National Key Universities), 396 teachers (54.5%) from provincial key institutions, and 53 teachers (7.3%) from vocational and technical institutions. Regarding the regions of the universities, 196 (27%) teachers were from universities in southern China, 131 (18%) were from those in eastern China, 65 (8.9%) were from those in northern and northeastern China, and 329 (45.3%) from those in western and central China. The average age of the participants was 37.65 years (*SD* = 7.77). Nearly all participants (99.7%) obtained a bachelor’s degree or higher. Of the participants, 461 (63.4%) obtained a PhD degree. Regarding teaching experience, 41% of the participants had < 5 years of teaching experience; 23.5%, 5–10 years; 24.2%, 11–20 years; and 11.3%, > 20 years. Of the participants, 13.3% were senior research assistants, 46.6% were lecturers/assistant professors, 29.3% were associate professors, and 10.7% were professors.

### Measures

Psychological detachment from work during nonwork time was assessed using the psychological detachment subscale of the Chinese version of the Recovery Experience Questionnaire [6, 45], which has acceptable reliability in the Chinese population [11, 26]. This scale comprises four items; the responses are rated on a 5-point Likert scale ranging from 1 (*strongly disagree*) to 5 (*strongly agree*). Some example items are as follows: “I distance myself from my work” and “I do not think about work at all.” A higher mean score indicates a higher level of psychological detachment. In the present study, Cronbach’s α for this scale was 0.74.

Emotional exhaustion was measured using the emotional exhaustion subscale of the Chinese version of the Maslach Burnout Inventory–General Survey [46, 47]. This scale has five items; the responses are rated on a 5-point Likert scale ranging from 1 (*never*) to 5 (*always*). Some example items are as follows: “I feel emotionally drained from my work” and “I feel burned out from my work.” A high mean score indicates a higher level of emotional exhaustion. In the present study, Cronbach’s α for this scale was 0.95.

Self-compassion was measured using the Chinese version of the Self-Compassion Scale-Short Form (SCS-SF) [48, 49]. This scale comprises 12 item; the responses are rated on a 5-point Likert scale ranging from 1 (*almost never*) to 5 (*almost always*). Some example items are as follows: “When something upsets me, I try to keep my emotions in balance” and “When I feel inadequate in some way, I try to remind myself that feelings of inadequacy are shared by most people.” A higher mean score indicates a higher level of self-compassion. In the present study, Cronbach’s α for this scale was 0.81.

Depressive symptoms were assessed using the depression subscale of the Depression–Anxiety–Stress Scale [50, 51]. This scale comprises seven items; the responses are rated on a 4-point scale ranging from 1 (*did not apply to me at all*) to 4 (*applied to me very much*). Some example items are as follows: “I felt downhearted and blue” and “I felt that I had nothing to look forward to.” Higher mean scores indicate higher degrees of depressive symptoms. In the present study, Cronbach’s α for this scale was 0.89.

Covariates commonly used in studies on work stress and depressive symptoms in university teachers [52–55] were also controlled for in the present study. These covariates included demographic variables (i.e., age, sex, educational level) as well as variables related to profession (i.e., years of teaching, professional title, and concurrent administrative position), location (i.e., northern and northeastern China, eastern China, southern China, and western and central China), and university level (i.e., national key institutions, provincial key institutions, junior vocational and technical institutions).

### Data analysis

Structural equation modeling was performed (using Mplus 8.3) to examine the hypothesized model. Missing data were accounted for through full information maximum likelihood. The following model fit indices were used to evaluate the model fit adequacy: χ² statistic, root mean square error of approximation (RMSEA; <0.08), comparative fit index (CFI; >0.90), and standardized root mean square residual (SRMR; <0.08). First, the direct and indirect effects of psychological detachment on depressive symptoms were tested using bootstrapping with 5000 resamples. Second, we investigated the moderating role of self-compassion in the association between emotional exhaustion and depressive symptoms. Emotional exhaustion and self-compassion were centered around the mean. The main effect of emotional exhaustion on depressive symptoms and its interaction with self-compassion were analyzed. Third, simple slope analysis was performed to determine the conditional slope for the associations between emotional exhaustion and depressive symptoms at high and low levels of self-compassion (low level: *M* − ≥1 × *SD*; high level: *M* + ≥ 1 × *SD*).

## Results

The mean scores and standard deviations of the key variables and the correlations are presented in Table 1. Psychological detachment was positively associated with self-compassion and negatively associated with emotional exhaustion and depressive symptoms. Emotional exhaustion was positively associated with depressive symptoms and negatively associated with self-compassion. Self-compassion was negatively associated with depressive symptoms.

First, we investigated the indirect role of emotional exhaustion in the association between psychological detachment and depressive symptoms. The model fit the data well: χ² = 21.276, *df* = 5, RMSEA = 0.067 with 90% CI [0.039, 0.097], CFI = 0.939, and SRMR = 0.018. Psychological detachment was negatively associated with emotional exhaustion (β = −0.118, *SE* = 0.037, *p* = .001), which was positively associated with depressive symptoms (β = 0.524, *SE* = 0.028, *p* < .001). The indirect effect of psychological detachment on depressive symptoms via emotional exhaustion (β = −0.062, *SE* = 0.023, *p* = .008, 95%CI [− 0.110, − 0.018]).

**Table 1:** Descriptive statistics and correlations among key study variables

	*M*	*SD*	1	2	3	4
1. Psychological detachment	2.75	0.75	—			
2. Emotional exhaustion	3.52	1.23	− 0.12^**^	—		
3. Self-compassion	3.30	0.50	0.16^**^	− 0.35^**^	—	
4. Depressive symptoms	1.53	0.57	− 0.07^*^	0.52^**^	− 0.53^**^	—

*Note*. * *p* < .05, ** *p* < .01, *** *p* < .001

**Fig. 2:**
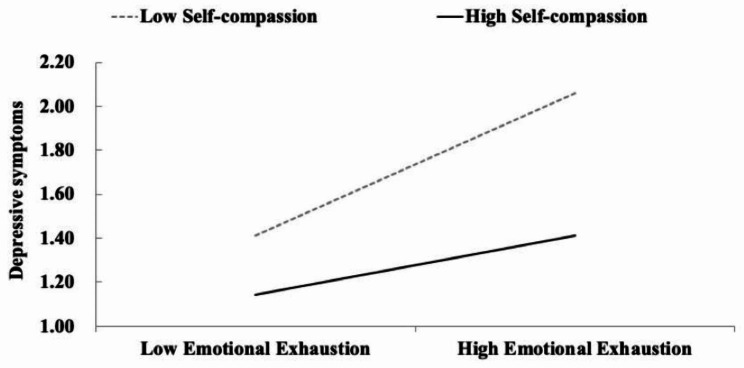
Plots depicting the interaction between emotional exhaustion and self-compassion

We investigated the moderating role of self-compassion in the association between emotional exhaustion and depressive symptoms. The model fit the data well: χ² = 21.402, *df* = 5, RMSEA = 0.067 with 90% CI [0.040, 0.098], CFI = 0.970, and SRMR = 0.017. The interaction between emotional exhaustion and self-compassion was negatively associated with depressive symptoms (β = –0.172, *SE* = 0.028, *p* < .001). The simple slope analysis revealed that the association between emotional exhaustion and depressive symptoms was stronger when the level of self-compassion was low (β = 0.523, *SE* = 0.035, *p* < .001) than when this level was high (β = 0.217, *SE* = 0.042, *p* < .001; Fig. 2). Overall, self-compassion alleviated the effect of emotional exhaustion on depressive symptoms. The indirect effects of psychological detachment on depressive symptoms through emotional exhaustion were found at both high and low levels of self-compassion (high level: β = −0.026, *SE* = 0.011, *p* = .023, 95% CI = [− 0.054, − 0.008]; low level: β =−0.062, *SE* = 0.023, *p* = .009, 95% CI = [− 0.111, − 0.018]).

## Discussion

In this study, we investigated the indirect role of emotional exhaustion in the association between psychological detachment and depressive symptoms and the moderating role of self-compassion. Our findings clarified how psychological detachment contributes to reducing depressive symptoms through an emotional pathway and how the emotion regulation strategy of self-compassion moderates this process. Psychological detachment and self-compassion work together to alleviate the adverse effect of emotional exhaustion on depressive symptoms. Consistent with the results obtained using the recovery–engagement–exhaustion model, which emphasizes the mediating role of exhaustion in the associations between recovery experiences and health outcomes [10], our findings suggest that psychological detachment from work during nonwork hours was negatively associated with depressive symptoms through emotional exhaustion. This finding corroborates those of studies reporting the effects of psychological detachment on affective state and depressive symptoms [2, 8, 15, 56], indicating that these effects can be noted in university teachers.

Our finding reveals that emotional exhaustion is a stress-related, emotional, and social problem that induces or exacerbates depressive symptoms [28]. Emotional exhaustion caused by work stressors depletes individuals’ energy and impairs mental health (e.g., depressive and anxiety symptoms) [34, 57]. With a positive function similar to those of other resources (e.g., social support), psychological detachment serves as a personal resource and disrupts the harmful relationship between emotional exhaustion and mental health [58]. Teachers who can “turn off” thoughts related to job and “switch off” mentally from job during nonwork time are more likely to enjoy the life and recovery from their leisure time [15, 24], which helps them to better deal with emotional exhausted and depressive state.

We identified a moderating role of self-compassion in the association between emotional exhaustion and depressive symptoms; this finding supports those of studies indicating that self-compassion is an effective emotion regulation strategy buffering the negative effect of emotional strain on depressive symptoms [42, 43]. In university teachers with higher levels of self-compassion, the association between emotional exhaustion and depressive symptoms was weaker. When self-compassionate teachers face emotional exhaustion resulting from work stress, they may exhibit resilience, treat themselves with care, accept their situation as a shared experience in educational settings, and maintain emotional stability [59, 60]. Thus, these teachers are able to protect their mental health from impairment due to emotional exhaustion.

The significant indirect effect of psychological detachment and the moderating effect of self-compassion indicate that these two psychological resources may work together to alleviate the harmful effect of emotional exhaustion caused by high levels of work stress. Detaching from work helps university teachers recover during leisure time, which contribute to reducing their subsequent exhaustion [7, 9], and self-compassion helps them to alleviate the detrimental effect of emotional exhaustion on depressive symptoms [41]. Our findings indicate that psychological detachment and self-compassion protect mental health by weakening the negative emotional pathway and affect-based mechanism involved in emotional exhaustion.

### Implications and limitations

Our findings have several implications. First, our model responds to calls for exploring the mechanism through which recovery experiences affect health outcomes and identification of how and for whom psychological detachment is the most beneficial [10, 27]. Our study expands the literature on the positive effects of psychological detachment and self-compassion in the contexts of work and education [15, 41]. By incorporating the recovery–engagement–exhaustion model and emotion regulation theory [10, 20], we identified the indirect role of emotional exhaustion and the moderating role of self-compassion. Our findings indicate that addressing psychological detachment and self-compassion can be an effective recovery strategy to deal with emotional strain resulting from work and protect mental health from impairment due to occupational stress. Universities should develop environments with restorative qualities (e.g., fascination and compatibility) for university teachers, which may help them better recover from work [27]. Our findings also suggest that the interplay between emotional pathway and emotion regulation strategies and their effects on depressive symptoms should be considered when exploring the positive effects of personal resources on mental health. This study provides insights into work stress management and mental health improvement programs. Because our findings demonstrate a combined weakening effect of psychological detachment and self-compassion on the adverse effect of emotional exhaustion on depressive symptoms, integrated interventions can encourage detachment from work (e.g., boundary management and engagement in recovery activities) and self-compassion (e.g., mindful self-compassion training) and combine these to reduce the emotional strain and distress that result from work stress [61, 62].

This study has several limitations. First, the present study could not investigate the longitudinal associations between key variables because of the cross-sectional design. Future studies should investigate causal relationships and the underlying moderated mediation mechanism by adopting longitudinal and experimental designs; in addition, within-person designs (e.g., daily diary and experience sampling approaches) should be adopted to examine within-person differences in the occurrence and intensity of recovery experiences and how these differences affect depressive symptoms [21]. Second, we only examine an emotion-based mechanism and emotion-related buffer in the association between psychological detachment and depressive symptoms. Nonetheless, other underlying mechanisms (e.g., fatigue) and moderators can influence the association between psychological detachment and outcomes [17]. Future studies should investigate these mechanisms and the protective role of other personal resources in the associations between psychological detachment and other outcomes (e.g., personal and work-related outcomes) [21]. Third, similar to most psychological detachment studies, the present study examines the associations between key variables in a multiorganizational convenience sample of Chinese university teachers; this limits the generalizability of our findings to other populations, contexts, and cultures. In the future, our model should be examined through additional systematic sampling and by considering the contextual and cultural influences. Finally, the present study is in line with most recovery studies relying on self-report data, which raises concerns regarding potential self-report bias and common method variance [63]. Future studies should measure strain and related outcomes by using cardiovascular and endocrinological indicators (e.g., cortisol levels and heart rate) [27].

## Data Availability

The datasets generated and/or analysed during the current study are not publicly available due to ethical issues but are available from the corresponding author on reasonable request.

## Declarations

This manuscript is not under review elsewhere and the results have not been published previously or accepted for publication. This manuscript has been seen and approved by all authors.
